# Antecedents of entrepreneurial networking behavior and its impact on business performance - a systematic literature review

**DOI:** 10.12688/f1000research.150032.1

**Published:** 2024-07-12

**Authors:** Sheetal Singh, Savitha Basri

**Affiliations:** 1 Department of Commerce, Manipal Academy of Higher Education, Manipal, Karnataka, 576104, India

**Keywords:** Entrepreneurship, Motivation, Networking behavior, Personality, Social networking

## Abstract

**Purpose:**

The purpose of this paper is to review the existing research on the antecedents of entrepreneurial networking behavior namely motivational factors and personality factors and its impact on business performance.

**Design/methodology/approach:**

This study employs a systematic review methodology, adhering to PRISMA guidelines.. Using the SCOPUS database, the search involved Boolean operators to narrow down relevant articles published in English between 2000 and 2024. Following a meticulous screening process, 32 articles were selected for review after removing duplicates and excluding non-English articles. Methodological quality assessment ensuring focused research questions, precise subject selection methods, representative samples, and reliable measurement instruments was carried out.

**Findings:**

The results demonstrate that entrepreneurs who are self-reliant and extroverted have fewer partners, however, these traits positively impact networking activities. The Big Five personality traits predict business creation and success of ventures. Desire for financial gain, risk-taking propensity, self-confidence, and the need for achievement influences networking behavior.

**Implications:**

The insights given in this paper can be used for establishing valuable connections, investing in resources, and preparing effective strategies for businesses. Policymakers who aim to promote entrepreneurial behavior among experienced individuals must emphasize the motivations for starting a business and the role of personalities in harnessing and leveraging individual entrepreneurial expertise.

**Originality:**

The existing literature on antecedents of entrepreneurial networking Behavior and the relationship of these antecedents together with business performance is limited. Further, this review article also offers avenues for future researchers.

## 1. Introduction

The start-up ecosystem in India is the third largest in the world after the USA and China (Economic Survey 2022-2023), and the number of start-ups increased significantly over the decade with the support of several government initiatives such as streamlined procedures, the granting of tax breaks, access to affordable internet services, and an enormous domestic market. However, 90% of start-ups fail within the first five years as a consequence of a lack of innovation, dynamism, or poor product-market fit, lack of leadership, skills, or expertise, inadequate funds, or even an absence of mentorship ( Kalyanasundaram, 2018; Kalyanasundaram *et al*., 2020). In this scenario, the social networking of an entrepreneur can lead his or her business to the next level.

Social networking is crucial for connecting people in the age of social media and for gathering information, resources, and knowledge. Through networking, people and organizations can interact with others in the sector, exchange knowledge, and discover fresh perspectives ( Gloor *et al*., 2018). Additionally, it might lead to prospects for funding, partnerships, and employment. Attending conferences, taking part in business events, joining professional groups, and making connections on social networking sites such as LinkedIn are just a few ways to build a network. It is important to develop and maintain relationships over time since networking involves more than just making connections, it is also about building long-lasting relationships. While performing social networking, entrepreneurs are often driven by their goals and aspirations, and it is the motivational factor that fuels their determination to succeed ( Abdallah *et al*., 2022). These factors can include personal ambition, a desire for financial success, the need for autonomy and independence, and the gratification derived from overcoming challenges. Research has shown that highly motivated entrepreneurs tend to have higher levels of persistence, resilience, and commitment to their business goals ( Murnieks *et al*., 2020). Similarly, understanding the role of the personality of an entrepreneur for social networking also plays a crucial role in determining business performance ( Di Stefano *et al*., 2023; Gloor *et al*., 2018). Research has shown that certain personality traits have a significant impact on an entrepreneur’s ability to succeed and perform well in their business ventures. These personality factors include traits such as extraversion, conscientiousness, openness to experience, emotional stability, and risk propensity ( Martin *et al.*, 2016).

The current review attempts to learn more about the antecedents of entrepreneurial networking behavior and its impact on Start-up performance. It also provides some suggestions for further study in this area. Many scholars have conducted systematic reviews of evidence on networking behavior, but a systematic review of the antecedents of networking behavior such as – motivational factors and personality factors, and their effects on business performance has not been conducted. Therefore, it is vital to examine existing research on the causes and consequences of networking behavior. The flow of this review article includes the literature review and its theoretical support, the methods used to identify all relevant articles, findings followed by discussions, suggestions for future research, and conclusions.

## 2. Literature review

### 2.1 Networking behavior

Social networking theory explains the complexities of networking by describing how social systems work among interconnected components ( Burt, 1997; Hatala, 2006). It is extensively used for measuring interpersonal dimensions of human interactions that explain how individuals choose different people for particular jobs ( Schultz-Jones, 2009). A network is a social structure that is larger than the sum of its parts and is composed of interpersonal relationships between a group of people ( Carter and Evans, 2006). Networking, in this context, is the process of developing, maintaining, and activating network relationships to improve the flow of information, capital, power, and friendship through these interactions ( Carter and Evans, 2006). In networking relationships, there may be exchanges of friendship, power, information, or goods as well as communication for mutual understanding and reciprocity to ratify trust. Interacting with others increases a person’s likelihood of gaining financial advantages, developing reputation, and establishing economic legitimacy ( Shane and Cable, 2002). Networking behavior is related to establishing close proximity to f business partners from whom an entrepreneur acquires business opportunities. Strong relationships are characterized by high level of trust, which enables social interactions and coordination. Weak ties, on the other hand, help to identify opportunities and are positively associated with benefits for the organization ( Elfring and Hulsink, 2003). More networking does not result in a large network, it depends on the networking talent of the founders and national cultural circumstances ( Baron, 2007). The size, diversity, density, openness, and stability of the entire network can be considered as the composition of a network ( Carter and Jones, 2006).

### 2.2 Motivational factors

Motivational factors are closely related to business growth and are consistent with prior research in social psychology and applied psychology ( Shane *et al.*, 2003). According to Sharafizad and Coetzer (2016), the motivation for beginning a firm can affect business strategy and operations while practicing networking. Men and women network differently, motivated by financial motivation, risk-taking, and self-confidence, with men scoring higher than women and having a stronger desire to build a firm ( Carter and Jones, 2006; Coleman, 2007). The need for achievement is also considered the main motivation for growing the business. The desire for independence is another motivational factor that encourages business owners to be their bosses, which has a favorable impact on the growth of their business. The desire for wealth has also been classified as a pull factor, although it may not be the driving force in the beginning ( DeMartino and Barbato, 2003; Alstete, 2003; Rosa and Dawson, 2006). In regard to wealth as a motivating factor, there are certain gender disparities ( Demartino and Barbato, 2003; Wilson *et al*., 2004). Baum et al. (2004) discovered that a diverse but interconnected set of motivational qualities and notions (along with talent) influence the performance of businesses. Furthermore, motivational factors can influence an entrepreneur’s decision-making process and ability to take risks. Entrepreneurs who are motivated to achieve success are more likely to set ambitious goals for their businesses, seize entrepreneurial opportunities, and actively pursue strategies for expansion and innovation ( Ismail, 2022). Entrepreneurial action can be influenced by the networking style of an entrepreneur, but this action can be changed according to multiple logics, including causality and effectuation approaches ( Sarasvathy, 2001; Fisher, 2012). Entrepreneurs believe that being socially enabled can benefit not only themselves but also their businesses. The greater the strength of cause or effect, the more motivated entrepreneurs are to participate in networks, particularly when evaluating the benefits to their businesses.

### 2.3 Personality factors

Personality may have a large impact on social networks and business success ( Jack *et al*, 2008). The Big Five personality traits are often considered the most comprehensive and accurate specifications of a personality ( Holt *et al.*, 2007; McCrae, 2011). Extraversion, agreeableness, openness to experience, conscientiousness, and neuroticism are the five basic elements that this model uses to determine personality. These personality traits are relevant for business studies since they reveal actions that are indicative of entrepreneurial ability ( Holt *et al.*, 2007; Obschonka *et al.*, 2012; Zheng *et al*., 2010). The Big Five personality model is useful for forecasting entrepreneurial business performance ( Martin *et al*., 2016). Several studies have found that higher levels of extraversion are positively associated with business performance. More extroverted entrepreneurs tend to be outgoing, sociable, and assertive, which can help them in networking, building relationships with customers and stakeholders, and confidently promoting their business ( Baluku *et al*., 2016; Kerr *et al*., 2018). Conscientiousness is another personality trait that is positively associated with business performance among entrepreneurs. Entrepreneurs who are highly conscientious tend to be diligent, organized, and responsible, which can contribute to better planning, time management, and task completion ( Jawabri, 2020). Openness to experience has been linked to business performance among entrepreneurs. Entrepreneurs who are open to new ideas, experiences, and opportunities are more likely to adapt and innovate in a rapidly changing business environment ( Kerr *et al*., 2018). This can lead to a competitive advantage, as they are more willing to explore new markets, products, and strategies. Emotional stability, or low neuroticism, is also important for business performance among entrepreneurs. Emotionally stable entrepreneurs are better able to handle stress, setbacks, and challenges that come with running a business. They are more resilient and capable of maintaining a positive mindset, which can help them navigate difficult situations and make sound decisions ( Kerr *et al*., 2018; Smith, 2013). Additionally, risk propensity influences entrepreneurial intentions but not necessarily entrepreneurial performance ( Baluku *et al*., 2016). This resilience enables them to stay focused and motivated, make rational decisions, and maintain a positive mindset, even in difficult circumstances. 

## 3. Methods

### 3.1 Research design

This systematic review of quantitative and qualitative studies was designed and implemented following the PRISMA framework ( Moher *et al*., 2009). The researchers used the PRISMA model (Preferred Reporting Items for Systematic Reviews and Meta-Analysis) to establish the structure of the study selection for review. After removing duplicates, 3879 publications were retrieved from the SCOPUS electronic databases. Initially, the titles and abstracts of all studies were screened and assessed against the inclusion and exclusion criteria to determine which were relevant to the review. Overall, 32 out of 230 studies were included in the analysis.

**Figure 1.**
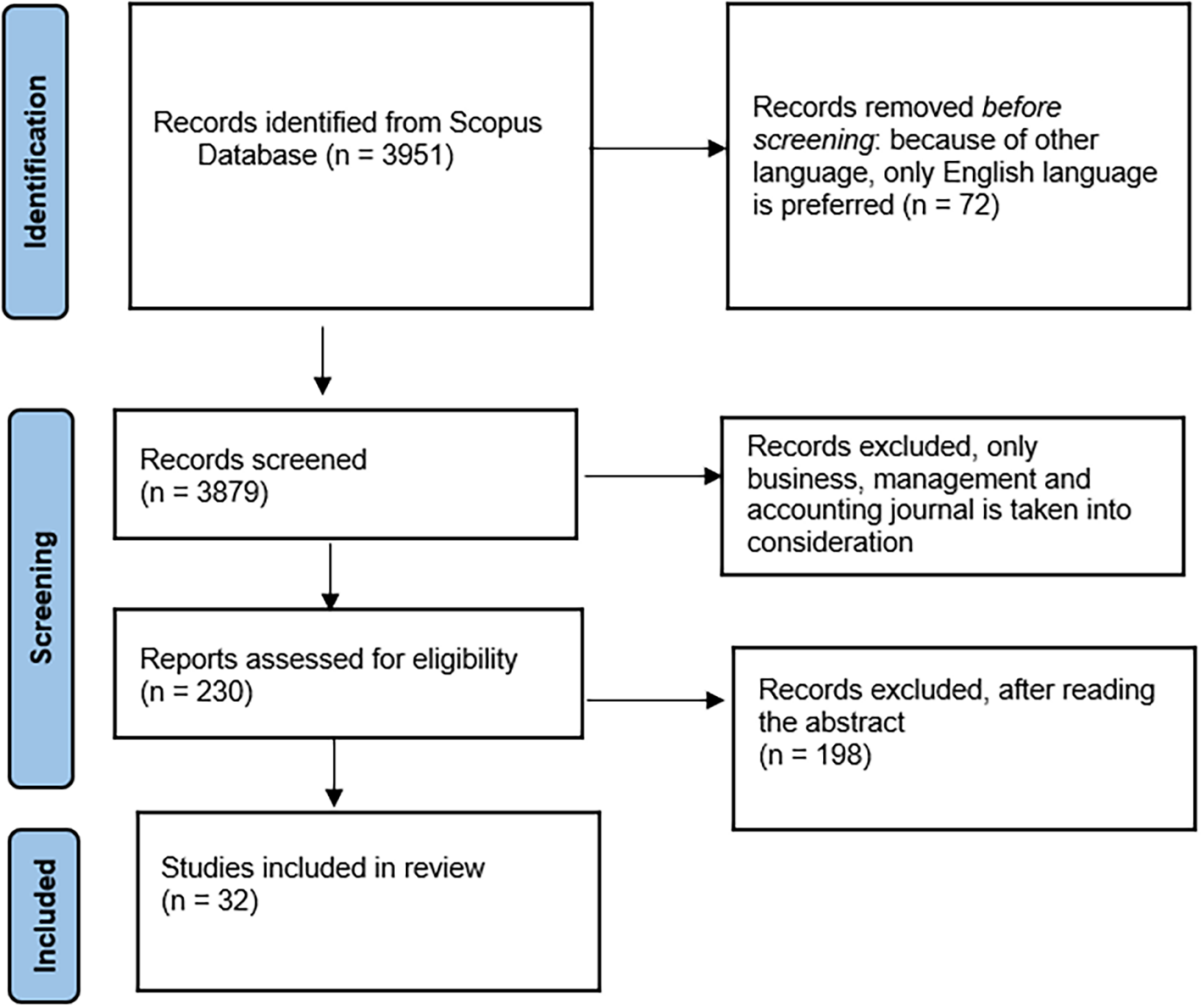
PRISMA flowchart.

The selection process is illustrated in the PRISMA flow chart presented in Figure 1 *.*

### 3.2 Search strategy and inclusion criteria

The SCOPUS database is used for a systematic search of relevant studies. The search strategies and terms were tailored to each database individually. Searches were performed by combining search terms using the Boolean operators AND, OR, and NOT. Searches were limited to peer-reviewed articles in English languages published between 2000 and 2024 to obtain an updated view of the research topic and to avoid overlapping results with the previous review *.* The iterative Booleans were utilized in various combinations in the search strategy namely, ‘networking behavior AND business performance’, ‘motivation AND business performance, and ‘personality AND business performance’, Microsoft Excel was used to create a data extraction sheet to reduce human error bias. Initially, 3951 papers were identified, and after determining duplication, 72 papers were considered for further screening. From the total of 230 studies, 32 studies were included for screening based on the research questions and their accessibility. Both longitudinal and cross-sectional research articles were chosen from the years 2000 to 2024, and these articles are mainly based on the English language.

### 3.3 Search outcome and exclusion criteria

230 articles remained for the title and abstract review ( Figure 1) *.* Articles retrieved based on abstracts were excluded from further analysis if they were not in English and irrelevant to the research questions. Reviews, editorials, and discussion papers also exclude the inclusion criteria. The researchers read the full texts of these articles to evaluate their eligibility for the review, resulting in the exclusion of a further 198 articles. The reason for exclusion of the studies did not address the research questions. Researchers screened every article independently. The 32 articles remaining after this process were selected for inclusion, and the researchers manually screened their references to identify additional relevant articles.

### 3.4 Quality assessment

The final data consisted of 32 quantitative and qualitative studies. All the studies included in the review addressed focused questions using appropriate research methods. Furthermore, the methods used to choose subjects has been clearly described. All the studies used representative samples and trustworthy measurement instruments. 

### 3.5 Data extraction and analysis

Specifying information about the publication, the purpose of the study, study subjects, study context, and significant findings reported in each article were extracted. The articles were analyzed by narrative synthesis ( Popay *et al*., 2006). Finally, only those 32 research papers that met the eligibility requirements were included in the study.

## 4. Overview of the reviewed studies

Table 1 shows the selected articles considered for the literature review in table format (refer to extended data table 1).

## 5. Findings and discussion

Most of the studies used a constructivist paradigmatic approach that was congruent with the methodologies used including case studies, narrative inquiry, ethnography, mixed-methods, and grounded theory. The majority of the studies have used a quantitative data approach to examine and comprehend entrepreneurs’ perceptions and actual networking behavior. The findings of these studies are significant for various reasons. It was found that most of the studies have been carried out in countries namely India, Slovenia, Uganda, Croatia, Germany, Portugal, and China. This may be because of the promotion of entrepreneurship as a tool for economic growth and unemployment reduction in developing nations, where entrepreneurs can access government incentives for their businesses to grow. The summary of review is given in Table 1.

Furthermore, it was found that the majority of studies used self-rating techniques, such as questionnaires which would have been biased toward some entrepreneurs because they might have given more weight to their positive attributes than to those that were essential for business success. Additionally, regarding personality as an antecedent to networking behavior, the majority of studies have examined only the Big Five traits to date, completely ignoring additional personality factors such as MBTI and nonpersonality factors namely cognition, knowledge, skills, and intelligence. The study sample sizes ranged from 50 to 3384 people. Critical events, content analysis, mixed approaches, embedded case studies, virtual experimental procedures, and in-depth personal interviews were all used in these studies, in addition to surveys. A small percentage of studies have used mixed techniques, with the majority of studies conducting only quantitative or qualitative research. The use of the mixed methods approach, which analyzes the evidence based on the current situation, may therefore be essential.

The process of building, cultivating, and mobilizing network links is known as networking. It includes the exchange of information, money, strength, and communication that occurs as a result of these ties ( Carter and Evans, 2006). Networks help in seeking information and advice, enriching internal resources, competing in flexible marketplaces, and creating advanced products ( Wiklund *et al*., 2009). Carter and Evans (2006) reported that large and complicated networks are more likely to provide business potential, increase problem-solving opportunities, and provide businesses with a greater chance of succeeding. Connecting with like-minded people, obtaining access to expertise and information, increasing learning, and building a sense of belonging and legitimacy are all major benefits of formal networking. Those who are interested in the success of a business use many networking practices ( Weber, 2007).

Motivational factors are closely related to business growth and are consistent with prior research in social psychology and applied psychology ( Shane *et al.*, 2003). According to Sharafizad (2019), networking should not be viewed as a simple stand-alone commercial activity, but rather as a complicated process that is influenced by the entrepreneur’s perceptions, and these are the reasons for founding the company first. The need for achievement is also considered the main motivation for growing the business. The desire for independence is another pull factor that encourages business owners to be their bosses, which has a favorable impact on the growth of their business. The desire for wealth has also been classified as a pull factor, although it may not be the driving force in the beginning ( DeMartino and Barbato, 2003; Alstete, 2003; Rosa and Dawson, 2006). Entrepreneurs are compelled to generate money, particularly in the development stage ( Alstete, 2003). In regard to wealth as a motivating factor, there are certain gender disparities ( Demartino and Barbato, 2003; Wilson *et al*., 2004). Baum *et al*. (2004) discovered that a diverse but interconnected set of motivational qualities and notions (along with talent) influence the performance of businesses. Entrepreneurial action can be influenced by the networking style of an entrepreneur, but this action can be changed according to multiple logics, including causality and effectuation approaches ( Sarasvathy, 2001; Fisher, 2012). While causal action stresses the choice of means for achieving the desired effect, effectual action highlights the potential consequences that can be achieved using the available means ( Sarasvathy, 2001). While entrepreneurial behavior emerges from commitments created in networks and connections from an effectuation perspective, networks can become a method for causal entrepreneurs to achieve the desired effect; entrepreneurs can choose whether to join these networks or not ( Fabris, 2021). Entrepreneurs believe that being socially enabled can benefit not only themselves but also their businesses. The greater the strength of cause or effect, the more motivated entrepreneurs are to participate in networks, particularly when evaluating the benefits to their businesses.

The Big Five personality traits are often considered as the most comprehensive and accurate specifications of a personality ( Holt *et al.*, 2007; McCrae, 2011). Extraversion, agreeableness, openness to experience, conscientiousness, and neuroticism are the five basic elements that this model uses to determine personality. These personality traits are relevant for business studies since they indicate actions that are indicative of entrepreneurial ability ( Holt *et al.*, 2007; Obschonka *et al.*, 2012; Zheng *et al*., 2010). Personality may have an enormous impact on social networks and have a direct impact on consequences ( Jack *et al*, 2008). Despite being a major indicator of entrepreneurial success, start-up capital accounted for only 2% of that success ( Baluku *et al*, 2016). The Big Five personality model is useful for forecasting entrepreneurial outcomes ( Baluku *et al*., 2016). The most important indicators of entrepreneurial success are agreeableness and extraversion. Agreeableness has an impact on the strength of network interactions. The findings of this study indicate that there are numerous components of a person’s social network, which are frequently characterized by a person’s personality. Some people may find it easier to form distinct social bonds and moderate the strength of the interactions than others ( Baluku *et al*, 2016).

Networking is a personal skill that entrepreneurs, particularly start-ups, require. A start-up can establish long-lasting relationships if the founder has effective communication skills and is well-known in the business world. As a result, business networking is critical for both new and established businesses. Networking gives business owners a competitive edge and gives them access to resources. The greatest outcome for entrepreneurs can be to access business opportunities, informational resources, more funds, and a greater consumer base which can be used to build and utilize partnerships with external stakeholders and organizations. ( Albourini *et al*., 2020) found that by cultivating external and internal contacts, entrepreneurs can improve the growth rate of their start-ups. Networking behavior leads to cultivating and nurturing relationships with other individuals and promoting future projects ( Forret and Dougherty, 2004). A rise in the customer base and better access to capital and information result from networking ( Albourini *et al*., 2020; Jack, 2005).

## 6. Summary and conclusion of the study

Although empirical research has dominated the field and covers a wide range of studies, there is no commonly acknowledged framework. This extensive research on the antecedents of entrepreneurial networking behavior and its impact on business performance has provided valuable insights into the complex dynamics that underlie the success of start-ups, particularly in countries such as Slovenia, Uganda, Sri Lanka, Germany, Portugal, and China. The findings from these studies have shed light on several critical aspects of networking behavior, personality traits, and their implications for entrepreneurial success. Motivational factors, such as the desire for financial gain, risk-taking propensity, self-confidence, and the need for achievement, play a crucial role in driving networking behavior among entrepreneurs. Understanding these motivations can help entrepreneurs align their networking efforts with their goals and strategies. The Big Five personality traits—extraversion, agreeableness, openness to experience, conscientiousness, and neuroticism—are significant predictors of entrepreneurial success. Entrepreneurs should recognize and leverage their unique personality traits to enhance their networking effectiveness. The frequency, reciprocity, and social interactions within a network are essential elements that can determine the quality and effectiveness of entrepreneurial networking. Entrepreneurs should focus on building meaningful interactions that foster trust and cooperation. Networking can lead to various positive outcomes, including access to business opportunities, information, funding, and a broader consumer base. Entrepreneurs who effectively cultivate and mobilize their network connections are more likely to achieve their business goals. Therefore, entrepreneurial networking is a multifaceted process influenced by motivations, personality traits, network structure, content, and interactions. Entrepreneurs who understand these factors and strategically harness the power of their networks are better positioned for success in the competitive world of start-ups. 

### 6.1 Limitations of the study and scope for further research

While these studies have provided valuable insights, there are still numerous avenues for future research. Most of the studies have used cross-sectional analysis, and future studies should undertake a longitudinal study to examine the evolving roles of the focal constructs in the model. There are very few studies adopting a qualitative approach for a specific industry, population group, or context. Therefore, a more qualitative approach may prove very insightful for understanding new industry segments and economic, and sociocultural contexts. The past working experience and cognitive abilities of entrepreneurs can also be studied to determine the differences in the networking behavior of naive and experienced entrepreneurs. Exploring the networking behavior of entrepreneurs in developed nations, considering additional personality factors beyond the Big Five, examining gender and experience effects on networking, and studying networking behaviors in different geographic settings (rural vs. metropolitan) are all areas that warrant further investigation.

### 6.2 Implications of the study

This research is important for entrepreneurs because networking behavior plays a crucial role in influencing business success. The insights given in this paper can be used for establishing valuable connections, investing in resources, and preparing effective strategies for businesses. Policymakers who aim to promote entrepreneurial behavior amongindividuals who have risk-taking propensity and the need for achievement motivation and emphasize the significance of harnessing and leveraging individual entrepreneurial personality dynamics through social networks.

## Author contributions

Sheetal Singh - Substantial contributions to the conception or design of the work, searching articles, conceptualization, first draft of the paper write-up.

Dr. Basri Savitha - Reviewing the manuscript critically for important intellectual content, writing the final manuscript, and editing the manuscript.

## Data Availability

No data are associated with this article. Figshare: networking among entrepreneurs. https://doi.org/10.6084/m9.figshare.25773411 ( Sheetal, 2024a). Figshare: PRISMA checklist for “Antecedents of entrepreneurial networking behavior and its impact on business performance - A systematic literature review”. https://doi.org/10.6084/m9.figshare.25710135 ( Sheetal, 2024b). Data are available under the terms of the Creative Commons Attribution 4.0 International license (CC-BY 4.0).
