# Supplementary material for: Antecedents of entrepreneurial networking behavior and its impact on business performance - a systematic literature review
Source: F1000Res. 2024 Jul 12;13:794. [Version 1] doi: 10.12688/f1000research.150032.1 (PMC11350331; doi:10.12688/f1000research.150032.1)
Supplement: Peer review history

# Reviewer report: Reviewer response for version 1

**DOI:** 10.5256/f1000research.164565.r315625

**Published:** 2024-08-27

**Authors:** Roshan Raj Bhujel

**Affiliations:** 1 Medhavi Skills University, Hee Burmiok, Sikkim, India

The paper "Antecedents of Entrepreneurial Networking Behavior and its Impact on Business Performance - A Systematic Literature Review" by Sheetal Singh and Savitha Basri is well-researched, offering a comprehensive and methodologically rigorous review of the existing literature. The study effectively utilizes PRISMA guidelines, ensuring a thorough and reliable analysis of how personality traits and motivational factors influence entrepreneurial networking behavior and business performance.

 Strengths:

 Methodological Rigor: The authors' systematic review, guided by PRISMA, is commendable for its clarity, thorough search strategy, and rigorous screening process, enhancing the reliability of the findings.

 Comprehensive Coverage: The review covers a broad time frame (2000-2024) and focuses on relevant literature, contributing to a well-rounded understanding of the topic.

 Key Findings: The identification of personality traits such as self-reliance, extroversion, and the Big Five traits, along with motivational factors like financial gain and risk-taking, provides valuable insights into entrepreneurial success.

 Practical Implications: The paper offers actionable recommendations for entrepreneurs and policymakers, emphasizing the importance of networking and strategic resource investment.

 Original Contribution: By addressing gaps in the literature and suggesting future research directions, the paper adds significant value to the field.

 Areas for Improvement: Clarification of Concepts: A brief, more detailed explanation of how the identified factors interact to influence business performance could further enhance understanding.

 Conclusion: This paper is a valuable contribution to the study of entrepreneurial networking behavior and its impact on business performance. The systematic review, comprehensive analysis, and practical implications make it worthy of acceptance.

Are the rationale for, and objectives of, the Systematic Review clearly stated?

Yes

Is the statistical analysis and its interpretation appropriate?

Not applicable

If this is a Living Systematic Review, is the ‘living’ method appropriate and is the search schedule clearly defined and justified? (‘Living Systematic Review’ or a variation of this term should be included in the title.)

Not applicable

Are sufficient details of the methods and analysis provided to allow replication by others?

Yes

Are the conclusions drawn adequately supported by the results presented in the review?

Yes

Reviewer Expertise:

This paper has key implications for policy and practice by highlighting how personality traits and motivations influence entrepreneurial networking and business success.Policy Implications: Policymakers can use these insights to design targeted support programs and incentives that align with traits like self-reliance and motivations such as financial gain, encouraging more effective networking among entrepreneurs.Practical Implications: Entrepreneurs can apply these findings to tailor their networking strategies based on their personality traits, while business educators can incorporate these insights into training programs to better prepare aspiring entrepreneurs for success.

I confirm that I have read this submission and believe that I have an appropriate level of expertise to confirm that it is of an acceptable scientific standard.

# Reviewer report: Reviewer response for version 1

**DOI:** 10.5256/f1000research.164565.r302646

**Published:** 2024-08-05

**Authors:** Doraiswamy Ashok

**Affiliations:** 1 Department of General Management, Vellore Institute of Technology, Vellore, India

This paper reviews the existing research on the antecedents of entrepreneurial networking behaviour, namely motivational factors and personality factors and its impact on business performance.

 This study employed a systematic review methodology adhering to PRISMA guidelines. Using the SCOPUS database, the search involved Boolean operators narrowing down relevant articles indexed in English between 2000 and 2024. The researcher has justified the period of review process on all possible materials up to a satisfactory level. The flow of this review article includes the literature review and its theoretical support, the methods used to identify all relevant articles, findings followed by discussions, suggestions for future research, and conclusions. Literature review coverage about networking behaviours, motivational factors and personality factors were mentioned in the highlights of theoretical models.   

 In the method session of the article, the model of PRISMA has been demonstrated step by step with the appropriate number of articles reviewed. Thus, the search criteria have proved satisfactory. 

 The findings and discussions were made with available insights from reviews used for the study purpose. Over all, the objective of the paper has served the purpose of demonstrating the antecedents of entrepreneurial networking behaviour.    

 “I have read this submission. I believe that I have an appropriate level of expertise to confirm that it is of an acceptable scientific standard.” Therefore it is approved for further consideration. 

Are the rationale for, and objectives of, the Systematic Review clearly stated?

Yes

Is the statistical analysis and its interpretation appropriate?

Partly

If this is a Living Systematic Review, is the ‘living’ method appropriate and is the search schedule clearly defined and justified? (‘Living Systematic Review’ or a variation of this term should be included in the title.)

Yes

Are sufficient details of the methods and analysis provided to allow replication by others?

Yes

Reviewer Expertise:

Management, Marketing, and Entrepreneurship

I confirm that I have read this submission and believe that I have an appropriate level of expertise to confirm that it is of an acceptable scientific standard.
